# Hip arthroscopy: a patient education and clinical research tool

**DOI:** 10.1093/jhps/hnv032

**Published:** 2015-05-16

**Authors:** Austin V. Stone, Allston J. Stubbs

**Affiliations:** Department of Orthopaedic Surgery, Wake Forest School of Medicine, Winston-Salem, NC 27157-1070, USA

Many mobile medical applications are available for the practicing surgeon, but few offer simultaneous clinical utility and hip specificity for the hip preservationist. The application, *Hip Arthroscopy* (Free on the AppStore for iPAD iOS), was developed by Alberto Sanchez, MD, PhD to meet the need for mobile software that comprises patient education and outcomes tools. This application provides point of care learning and clinical outcomes resources- primarily on femoroacetabular impingement (FAI). The application is available in English and Spanish, and may be downloaded by both patients and practitioners.

The diagnosis of FAI is explained in a detailed patient brochure accessible from the application’s home screen. The brochure contains simple but informative diagrams of hip anatomy and the pathomechanics of FAI. The language is concise and accessible to the lay audience. A basic description of the pathology is followed by expectations for the clinical exam and possible treatment options. Treatment options include both non-operative and operative management plans. A video demonstrating the mechanics FAI aids in patient understanding. The application may be accessed by patients from home or recommended for review after a clinic appointment.

Clinical utility of the application is enhanced by the pre-loaded outcomes questionnaires. These include the following: iHOT-12 (International Hip Outcome Tool, short version) and iHOT-33 (long version), HOS (Hip Outcome Score), HOSS (Hip Outcome Score Sports), NAHS (Non Arthritic Hip Score), mHHS (Modified Harris Hip Score) and WOMAC (Western Ontario and McMaster Universities Arthritis Index). The Visual Analog Score for pain is also available. The user interface is simple and rapid; e.g. the iHOT-33 can be completed in <3 min. These outcomes questionnaires can be emailed from the device to print, transfer into a database, or upload into an electronic medical record. The application has the capacity to reduce research costs and increase outcomes reporting by permitting data collection in the clinic or from any iOS device running the software.

Although the application has great potential for a hip preservation practice, there are several limitations. First, integration across the clinical and research data entry points is limited. Second, the user interface for surgical documentation is not immediately intuitive and does not flow to other aspects of the program. Next, the utility of special functions such as radiographic measurement is unique, but requires several steps beyond a typical digital radiology interface. Finally, there is no security or encryption function that restricts transfer of private health information across unsecure networks.

In summary, practitioners and researchers will find value in this application for recording both subjective outcome scores and objective clinical data. Further, the educational component of the program offers patients a simple and responsive interface on hip arthroscopy and FAI. Despite certain limitations, *Hip Arthroscopy* has the advantage of efficiency in data collection and mobility.

